# Changes in Added Sugar Intake and Body Weight in a Cohort of Older Australians: A Secondary Analysis of the Blue Mountains Eye Study

**DOI:** 10.3389/fnut.2021.629815

**Published:** 2021-03-01

**Authors:** Hanieh Moshtaghian, Karen E. Charlton, Jimmy Chun Yu Louie, Yasmine C. Probst, Paul Mitchell, Victoria M. Flood

**Affiliations:** ^1^School of Medicine, Faculty of Science, Medicine and Health, University of Wollongong, Wollongong, NSW, Australia; ^2^Illawarra Health and Medical Research Institute, Wollongong, NSW, Australia; ^3^School of Biological Sciences, Faculty of Science, The University of Hong Kong, Pokfulam, Hong Kong; ^4^Center for Vision Research, Department of Ophthalmology and Westmead Millennium Institute, University of Sydney, Sydney, NSW, Australia; ^5^Sydney School of Health Sciences, Faculty of Medicine and Health, University of Sydney, Sydney, NSW, Australia; ^6^Westmead Hospital, Western Sydney Local Health District, Westmead, NSW, Australia

**Keywords:** added sugar intake, added sugar food sources, body weight, Blue Mountains Eye Study, older adults, cohort study

## Abstract

**Background:** The evidence regarding the association between added sugar (AS) intake and obesity remains inconsistent. The aim of this study was to investigate the association between changes in the percentage of energy intake from AS (EAS%) and changes in body weight in a cohort study of older Australians during 15 years of follow-up. In addition, associations were assessed according to whether EAS% intake was provided from beverage or non-beverage sources.

**Methods:** Data were analyzed from the participants of the Blue Mountains Eye Study Cohort. Dietary data were collected at baseline (1992–94) and three five-yearly intervals using a 145-item food frequency questionnaire. Participants' body weight was measured at each time point. Five-yearly changes in EAS% intake and body weight were calculated (*n* = 1,713 at baseline). A generalized estimating equation (GEE) model was used to examine the relationship between the overall five-yearly changes in EAS% intake and body weight, adjusted for dietary and lifestyle variables.

**Results:** In each time interval, the EAS% intake decreased by ~5% in the lowest quartile (Q1) and increased by ~5% in the highest quartile (Q4). The mean (SD) body weight change in Q1 and Q4 were 1.24 (8.10) kg and 1.57 (7.50) kg (first time interval), 0.08 (6.86) kg and −0.19 (5.63) kg (second time interval), and −1.22 (5.16) kg and −0.37 (5.47) kg (third time interval), respectively. In GEE analyses, the overall five-yearly change in EAS% intake was not significantly associated with body weight change (*P*_trend_ = 0.837). Furthermore, no significant associations were observed between changes in EAS% intake from either beverage or non-beverage sources and changes in body weight (*P*_trend for beverage sources_ = 0.621 and *P*_trend for non-*beverage sources*_ = 0.626).

**Conclusion:** The findings of this older Australian cohort do not support the association between changes in EAS% intake and body weight, regardless of AS food sources (beverage or non-beverage).

## Introduction

Obesity is a major public health issue in all age groups, including the elderly. Several physical and mental health complications (e.g., cardiovascular disease and depression) are associated with obesity in this population (1). Obesity has a negative impact on the overall quality of life and chronic disease-free life expectancy of older adults (2). Therefore, there is a global focus on the prevention of obesity to reduce the financial and psychosocial burden of the obesity-associated chronic diseases, particularly among the older sectors of the population (3–5). Of the multifactorial causes of obesity, diet is a modifiable risk factor and a target for many weight loss programs (6).

Diets high in energy-dense foods have been linked to obesity (7), and added sugar (AS) has been identified as a major component in many energy-dense foods (8). AS is defined as sugars and syrups added to foods during processing and preparation (9). Honey and concentrated fruit juices used as an ingredient in the multi-ingredient foods are also considered to be AS (10). This is similar to the World Health Organization free sugar definition where all monosaccharaides and disaccharides added to foods (by the manufacturer, cook, or consumer) and sugars naturally present in honey, syrups, fruit juices, and fruit juice concentrates are considered in the free sugar definition (11).

Several intervention studies have investigated the effect of AS intake on the weight gain or development of obesity. These studies accounted for individual types of sugars (e.g., fructose or glucose) or specific food sources of AS (i.e., sugar-sweetened beverages) (12–15), but did not consider the contribution of total AS intake. In addition, some studies were short-term (<1 month) (12, 13, 15), therefore, the generalization of the outcomes of these studies to the general population and everyday diets is limited. Conversely, cohort studies with longer follow-up periods have tended to focus on sugar-sweetened beverages as major sources of AS. In most cases, these studies have reported a positive association between the intake of these beverages and indicators of obesity (16, 17). Nevertheless, the association has not been confirmed in all studies nor across all age groups, including older adults (18, 19).

Few cohort studies have been conducted to investigate the association between baseline AS intake and change in body weight or body mass index (BMI) (19, 20). A longitudinal study that investigated the change in AS intake in children and adolescents reported no significant associations between AS intake, or AS intake from liquid or solid food sources, and BMI when the analysis was adjusted for total energy intake (21). This was contrary to findings from other cohort studies, in which increases in the intake of sugar sweetened beverages were associated with an increased BMI (22, 23). In addition to inconsistent findings for the liquid food sources of AS, results of studies on the association between other AS food sources (e.g., sweets, desserts, and other solid sources) and obesity indicators (24, 25) were also contradictory.

To our knowledge, the longitudinal association between change in AS intake and body weight change over time has not been investigated in adults, including older populations. The aim of the current study is, therefore, to investigate the associations between change in the percentage of energy intake from AS (EAS%) and body weight change in a longitudinal cohort of older Australians during 15 years of follow-up. In addition, associations were assessed according to whether EAS% intake was provided from beverage or non-beverage sources.

## Methods

### Study Population

This study is a secondary analysis of data from the Blue Mountains Eye Study (BMES) cohort. Details of the BMES have been described in detail elsewhere (26). Briefly, participants of the BMES were aged ≥49 years old at baseline and lived in two postcode areas within the Blue Mountains region, New South Wales, Australia. Baseline data collection (BMES 1) was conducted between 1992 and 1994 and subsequent data were collected every 5 years (BMES 2:1997–99, BMES 3:2002–04, and BMES 4:2007–09). The BMES has ethics approval from the Sydney West Area Health Services and the University of Sydney Human Research Ethics Committees. Written informed consent was obtained from all participants.

### Dietary Data Collection

Dietary data were collected using a validated 145-item semi-quantitative food frequency questionnaire (FFQ) (27) and analyzed using corresponding food composition data from the Australian Nutrient Tables (NUTTAB) to assess nutrient intakes. NUTTAB 1990 was used for the BMES 1, NUTTAB 1995 for BMES 2 and 3 and NUTTAB 2010 for BMES 4, to align with the food supply at each follow up time-points. The NUTTAB databases contain the nutrient content of various foods, including total sugars, but do not contain AS values. Therefore, the AS content of FFQ food items was estimated for each NUTTAB using a stepwise method (28). In this 10-step systematic method, foods with zero total sugar and natural/unprocessed foods were considered to have zero AS. Foods containing 100% AS (i.e., no naturally occurring sugar), such as regular soft drinks, were considered to have an AS content equivalent to the total sugar content (28). The AS content of other foods was estimated from recipes, comparisons of the total sugar content of sweetened products with unsweetened varieties, analytical data for individual sugar types (i.e., lactose and maltose), adoption of values from other countries' AS databases or using food labels (28).

FFQ dietary intake data were cleaned for implausible energy and nutrient intakes (26). For example, FFQs with 12 blank questions or blank page(s) were excluded. Participants with implausible energy intakes of <2,500 kJ and >18,000 kJ or extreme AS intakes of more than the mean ± 4 Standard Deviation (SD) were also excluded. For this study, only participants who provided both dietary and body weight data at two consecutive time points (paired observations) were included in the analyses. These paired observations were provided by 1,713 participants in the first time interval (BMES 1–2), 1,209 participants in the second time interval (BMES 2–3) and 747 participants in the third time interval (BMES 3–4). Thus, the total number of paired observations were 3,669. Throughout this study, the beginning of each time interval is referred to as the “initial” time point.

### Classification of Beverage vs. Non-beverage Added Sugar Sources

Food groups were developed (29) based on the 1995 Australian National Nutrition Survey (NNS1995) (30). For AS analyses, food groups were modified to represent AS food groups (31) and were then classified to beverage (liquid) or non-beverage (non-liquid: semi-solid/solid) categories. The beverage category included the sugar-sweetened beverages group and was formed based on the definition of sugar-sweetened beverages. According to this definition, water-based non-alcoholic beverages containing AS, including regular soft drinks, cordial, electrolyte and energy drinks, fruit, and vegetable drinks (excluding milk, 100% fruit juice, and artificially sweetened drinks) are considered sugar-sweetened beverages (32, 33). Thus, in this study, the beverage category included sugar-sweetened beverages (sweetened juices, cordial and soft drinks) and the non-beverage category included cereal products (breakfast cereals), cereal-based products and dishes (biscuits, cakes, buns and scones, pastries, and mixed dishes), dairy products and dishes (yogurt, custard, ice cream, and dairy-based desserts), sugar products and dishes (discretionary sugar, honey, jam, and syrup), confectionary (sweets and chocolate), savory sauces, meat (processed), and vegetables (processed/canned varieties).

### Assessment of Body Weight and Covariates

For the BMES anthropometry assessments, participants' body weight (kg) was measured with electronic scales (without shoes or heavy clothes) (34, 35) at each time point. Since physical stature declines in aging populations (36), change in weight over time was used as an outcome rather than change in BMI. For assessing the covariates, information was collected about the participants' medical history and socio-demographic and lifestyle factors by trained interviewers at all BMES time points using extensive questionnaires (37, 38). Some of these questions were about participants' smoking status (i.e., never smoked, past smoker, or current smoker), and physical activity (39). Physical activity questions collected information regarding walking exercises and moderate-to-vigorous activities over the previous 2 weeks (39). Metabolic equivalents (METs) over 1 week were calculated based on participants' responses to the questions (39), using the International Physical Activity Questionnaire scoring protocol (40). This information was used in the current study to adjust data for smoking and physical activity.

### Statistical Analyses

The changes in EAS% intake were reported as quartiles at each 5-year time interval (BMES 1–2, BMES 2–3, and BMES 3–4). The longitudinal associations between the overall 5-yearly changes in EAS% and changes in body weight during the 15 years of follow-up were investigated using a generalized estimating equation (GEE) model. The unstructured correlation matrix was selected for the GEE model to consider the within-individual correlations between the repeated observations for the same participant. In this longitudinal analysis, the first and last quartiles represented the largest decrease and the largest increase in EAS% intakes for each time interval, respectively. The change in EAS% intake was used as a continuous variable to assess *P* for trends.

The GEE analysis was adjusted for gender and the initial age of participants in each paired observation (Model 1). Further adjustments were made for the initial data for weight, diabetes status and EAS% intake, and both the initial and the changes in each time interval for the following variables: fiber intake, total energy intake, glycaemic index, physical activity, and smoking status (Model 2). Similar adjustment models were used for AS food source analyses by replacing EAS% intake variable with EAS% intakes from beverage and non-beverage sources. All analyses were performed in SPSS software (Version 21, SPSS Inc., Chicago, IL, USA) and statistical significance was set at *P* < 0.05.

## Results

### Participants Characteristics

The baseline characteristics of the BMES 1 participants are shown in Table 1. The mean age of BMES 1 participants was 63.8 years. The baseline mean EAS% intake was 9.40% (1.02% from beverage and 8.38% from non-beverage sources) and their mean BMI was 26.22 kg/m^2^. A higher proportion of the participants in the lowest quartile were smokers compared to those in other quartiles. Participants in quartile 1 had higher EAS% intakes and BMI compared to those in quartile 4. In addition, their EAS% intakes from beverage and non-beverage sources were higher than other participants. Baseline characteristics of BMES 1 participants who provided paired observations in BMES 2 vs. those without paired observations are presented in Supplementary Table 1. Participants with paired observations had a slightly lower EAS% intake compared to those without paired observations (9.40 vs. 10.14%), but their BMI was similar.

**Table 1:** Baseline characteristics of BMES participants according to quartiles of change in percentage of energy from added sugar (EAS%) intake during the first 5-year time interval (BMES 1–2).

	**Change in EAS% intake**				
	**All**	**Q1**	**Q2**	**Q3**	**Q4**
Median	0.22	−4.50	−0.83	1.51	5.19
*n*	1,713	428	428	429	428
Women (*n*, %)	978 (57.09)	227 (53.04)	226 (52.80)	256 (59.67)	269 (62.85)
Age, years	63.8 (8.2)	63.5 (8.6)	64.3 (7.9)	63.0 (8.1)	64.4 (8.2)
Current smoker (*n*, %)	199 (11.62)	66 (15.42)	43 (10.05)	40 (9.32)	50 (11.68)
Diabetes (*n*, %)	104 (6.07)	25 (5.84)	28 (6.54)	29 (6.76)	22 (5.14)
Married (*n*, %)	1,186 (69.24)	278 (64.95)	308 (71.96)	311 (72.49)	289 (67.52)
Qualification after leaving school (*n*, %)	1,040 (60.71)	248 (57.94)	269 (62.85)	265 (61.77)	258 (60.28)
Home ownership (*n*, %)	1,567 (91.48)	389 (90.89)	394 (92.06)	401 (93.47)	383 (89.49)
Living alone (*n*, %)	381 (22.24)	105 (24.53)	94 (21.96)	80 (18.65)	102 (23.83)
Energy, kJ	8,599 (2,481)	8,614 (2,675)	8,727 (2,494)	8,739 (2,345)	8,318 (2,385)
Fat, E%	32.82 (6.21)	32.44 (6.04)	32.97 (6.07)	32.90 (6.33)	32.96 (6.39)
Protein, E%	17.76 (3.08)	16.70 (2.86)	17.80 (2.77)	18.45 (2.99)	18.09 (3.39)
Alcohol, E%	3.81 (5.61)	3.22 (4.76)	4.11 (5.71)	4.26 (5.95)	3.65 (5.88)
Carbohydrate, E%	46.91 (7.64)	49.08 (7.13)	46.44 (7.27)	45.60 (7.93)	46.51 (7.80)
Added sugar, E%	9.40 (5.16)	13.75 (5.31)	8.93 (4.14)	7.16 (3.77)	7.76 (4.51)
Beverage AS, EAS%	1.02 (1.98)	1.83 (3.01)	0.75 (1.30)	0.61 (1.02)	0.88 (1.74)
Non-beverage AS, EAS%	8.38 (4.67)	11.93 (4.89)	8.18 (3.88)	6.54 (3.55)	6.87 (4.17)
Fiber, g	29.07 (12.00)	27.03 (12.06)	29.29 (11.11)	31.36 (12.75)	28.59 (11.66)
Glycaemic index	56.56 (4.34)	57.30 (3.93)	56.75 (4.30)	55.72 (4.23)	56.47 (4.72)
BMI, kg/m^2^	26.22 (4.28)	26.39 (4.56)	26.28 (4.37)	26.01 (4.18)	26.20 (4.00)
Body weight, kg	72.14 (13.51)	72.67 (14.09)	73.33 (13.93)	71.48 (13.10)	71.10 (12.80)
Physical activity, MET	1,446 (2,404)	1,367 (2,269)	1,428 (2,410)	1,544 (2,384)	1,442 (2,546)

*EAS%, percentage of energy from added sugar; AS, added sugar; BMI, body mass index; MET, metabolic equivalent*.

*All data were presented as mean (SD) except data for women, current smoker, diabetes, married, qualification after leaving school, home ownership and living alone, where data were presented as n (%)*.

### Changes in Added Sugar and Weight in Each Time Interval

Change in dietary intake and weight across quartiles of changes in EAS% intake at the first, second and third time intervals are presented in Table 2. The EAS% intake at 5-year intervals decreased by ~5% in the lowest quartile and increased by ~5% in the highest quartile. The mean of change in energy intake was <200 kJ in the lowest quartiles and <75 kJ in the highest quartiles. The median change in AS intake for the largest decrease (−4.85%) was −26.39 g in the third time interval and for the largest increase (5.19%) was 26.59 g in the first time interval.

**Table 2:** Mean (SD) changes (Δ) in macronutrient intake and body weight according to quartiles of change in percentage of energy from added sugar (EAS%) during three 5-year time intervals in the BMES cohort.

	**Change in EAS% intake**			
	**Q1**	**Q2**	**Q3**	**Q4**
**First time interval (BMES 1**–**2)**				
Median	−4.50	−0.83	1.51	5.19
*n*	428	428	429	428
ΔEnergy, kJ	−198 (2,622)	−223 (2,185)	40 (2,311)	53 (2,431)
ΔCarbohydrate (excluding AS), g	14.61 (63.02)	1.98 (53.46)	4.79 (61.70)	−8.21 (60.25)
ΔFat, g	−2.65 (29.42)	−4.66 (25.57)	−4.01 (26.76)	−5.08 (28.31)
ΔProtein, g	5.75 (29.39)	−0.14 (26.21)	−1.48 (26.72)	−4.41 (28.31)
ΔAlcohol, g	−0.05 (9.67)	−0.66 (11.28)	−0.42 (10.99)	−2.40 (11.76)
ΔBeverage AS, EAS%	−0.72 (2.63)	0.10 (1.23)	0.55 (1.37)	1.83 (3.39)
ΔNon-beverage AS, EAS%	−4.80 (3.50)	−1.01 (1.37)	0.98 (1.48)	4.55 (3.86)
ΔBody weight, kg	1.24 (8.10)	0.86 (8.03)	2.29 (6.12)	1.57 (7.50)
**Second time interval (BMES 2**–**3)**				
Median	−4.56	−1.13	1.08	4.71
*n*	302	302	303	302
ΔEnergy, kJ	93 (2,424)	431 (2,417)	127 (2,085)	−36 (2,265)
ΔCarbohydrate (excluding AS), g	11.21 (61.33)	9.13 (60.80)	−6.40 (53.37)	−15.57 (55.91)
ΔFat, g	4.79 (26.39)	5.16 (27.64)	4.59 (24.38)	−1.26 (27.03)
ΔProtein, g	8.45 (26.80)	7.95 (28.41)	−0.60 (26.84)	−5.03 (26.52)
ΔAlcohol, g	0.78 (9.37)	0.47 (10.54)	−1.19 (9.27)	−2.71 (10.71)
ΔBeverage AS, EAS%	−1.69 (3.26)	−0.26 (1.47)	0.07 (1.28)	1.31 (2.82)
ΔNon-beverage AS, EAS%	−4.06 (3.48)	−0.93 (1.60)	1.08 (1.37)	4.28 (3.50)
ΔBody weight, kg	0.08 (6.86)	0.84 (6.58)	0.60 (5.15)	−0.19 (5.63)
**Third time interval (BMES 3**–**4)**				
Median	−4.85	−1.19	0.96	4.57
*n*	186	187	187	187
ΔEnergy (kJ)	−94 (2,554)	−260 (2,206)	−4 (2,166)	74 (1,988)
ΔCarbohydrate (excluding AS), g	4.87 (61.14)	−11.88 (53.56)	−9.29 (57.69)	−18.62 (56.38)
ΔFat, g	2.99 (31.60)	−0.30 (26.55)	1.68 (24.28)	0.79 (24.67)
ΔProtein, g	9.14 (30.14)	1.43 (26.07)	0.67 (25.39)	−5.01 (25.35)
ΔAlcohol, g	−0.75 (7.68)	−0.08 (8.84)	−1.69 (10.86)	−1.71 (7.91)
ΔBeverage AS, EAS%	−1.22 (2.38)	−0.29 (1.14)	−0.05 (1.36)	1.12 (2.59)
ΔNon-beverage AS, EAS%	−4.50 (3.36)	−0.95 (1.29)	1.09 (1.41)	4.39 (3.72)
ΔBody weight, kg	−1.22 (5.16)	−1.76 (6.16)	−0.57 (4.99)	−0.37 (5.47)

*EAS%, percentage of energy from added sugar; AS, added sugar*.

In each time interval, participants in the lowest quartile who decreased their EAS% intake, increased their protein, other carbohydrates, fat or alcohol intakes; whereas participants in the highest quartile who increased their EAS% intakes decreased their protein, other carbohydrates, alcohol, or fat intakes. The weight gain occurred across all quartiles in the first and the second time intervals (except quartile 4 in the second time interval), and the mean body weight change in the lowest quartile was <1.3 kg and in the highest quartile was <1.6 kg. In the third time interval, those who increased their EAS% intake experienced less weight loss compared to those who decreased their intake, but weight loss occurred across all quartiles of change in EAS% intakes.

Changes in body weight across the quartiles of change in EAS% intake from beverage and non-beverage sources in each 5-year time interval are presented in Table 3. The largest increase in EAS% from beverage and non-beverage sources were 2.09 and 4.18% in the first time interval, respectively. The mean (SD) change in AS and energy intake for a 2.09% increase in EAS% from beverage sources were 15.92 (15.72) g and 224 (2,369) kJ, and for a 4.18% increase in EAS% from non-beverage sources were 24.10 (20.34) g and −35 (2,436) kJ, respectively (data not shown). For both AS sources, the mean of change in body weight in the first and fourth quartile was <1.8 kg. For EAS% intakes from non-beverage sources in the third time interval, those who had the highest increase in EAS% intake from non-beverage sources experienced less weight loss compared to those who decreased their EAS% intakes from these sources.

**Table 3:** Mean (SD) changes (Δ) in body weight according to quartiles of change in percentage of energy from added sugar (EAS%) from beverage and non-beverage sources during three 5-year time intervals in the BMES cohort.

	**Change in EAS% intake**			
	**Q1**	**Q2**	**Q3**	**Q4**
***ΔBeverage AS***				
**First time interval (BMES1-2)**				
Median	−0.71	0.00	0.32	2.09
*n*	428	428	429	428
ΔBody weight, kg	1.22 (7.94)	1.11 (6.99)	2.11 (7.87)	1.52 (7.10)
**Second time interval (BMES 2-3)**				
Median	−1.89	−0.18	0.06	1.37
*n*	302	302	303	302
ΔBody weight, kg	0.77 (7.43)	0.41 (5.96)	0.14 (5.25)	0.03 (5.53)
**Third time interval (BMES 3-4)**				
Median	−1.47	−0.21	0.02	1.09
*n*	186	187	187	187
ΔBody weight, kg	−1.13 (5.48)	−0.45 (5.36)	−1.11 (4.99)	−1.23 (6.05)
***ΔNon-beverage AS***				
**First time interval (BMES1-2)**				
Median	−4.40	−1.00	0.86	4.18
*n*	428	428	429	428
ΔBody weight, kg	1.01 (9.02)	1.25 (7.40)	1.96 (4.93)	1.74 (7.99)
**Second time interval (BMES 2–3)**				
Median	−3.92	−0.89	1.04	4.15
n	302	302	303	302
ΔBody weight, kg	−0.03 (6.69)	0.57 (4.66)	0.99 (7.26)	−0.19 (5.39)
**Third time interval (BMES3-4)**				
Median	−4.26	−0.83	1.09	3.78
*n*	186	187	187	187
ΔBody weight, kg	−1.69 (5.93)	−1.37 (5.25)	−0.70 (5.15)	−0.16 (5.48)

*EAS%, percentage of energy from added sugar; AS, added sugar*.

### Overall Changes in Added Sugar and Weight

The longitudinal analyses for the associations between the overall 5-yearly changes in EAS% intake and body weight during 15 years of follow-up are presented in Table 4. In both adjusted models, the association between change in EAS% intake and body weight change was not statistically significant (Model 1 *P*_trend_ = 0.079; Model 2 *P*_trend_ = 0.837). Similar results were observed for the association between changes in EAS% intake from either beverage or non-beverage sources and changes in body weight (*P*_trend_ = 0.621 and *P*_trend_ = 0.626, respectively). It is also worth noting that analyses in Model 2 were repeated by the exclusion of the initial and the changes in total energy intakes from the adjustment model and the results of these reanalyses provided similar findings.

**Table 4:** The overall 5-yearly change (Δ) in body weight according to quartiles of change in energy from added sugar intake (EAS%) in the BMES cohort during 15 years of follow-up.

	**Change in EAS% intake**^****a****^				**Estimate (95% CI)^**b**^**	***P*_**trend**_^**c**^**
	**Q1**	**Q2**	**Q3**	**Q4**		
	**Δ** **Body weight (kg: Mean [95% CI]) for change in EAS%**					
Model 1^d^	0.35 (−0.08, 0.78)	0.38 (−0.07, 0.82)	1.08 (0.74, 1.42)	0.70 (0.29, 1.12)	0.050 (−0.006, 0.105)	0.079
Model 2^e^	0.22 (−0.65, 1.08)	−0.05 (−0.85, 0.75)	0.51 (−0.31, 1.34)	0.15 (−0.81, 1.12)	0.006 (−0.048, 0.060)	0.837
	**Δ** **Body weight (kg: Mean [95% CI]) for change in EAS% from beverage sources**					
Model 1^d^	0.50 (0.05, 0.95)	0.66 (0.27, 1.06)	0.82 (0.41, 1.22)	0.53 (0.13, 0.92)	0.023 (−0.067, 0.112)	0.618
Model 2^f^	0.06 (−0.85, 0.97)	0.27 (−0.54, 1.08)	0.43 (−0.51, 1.37)	0.10 (−0.71, 0.91)	−0.028 (−0.141, 0.084)	0.621
	**Δ** **Body weight (kg: Mean [95% CI]) for change in EAS% from non**-**beverage sources**					
Model 1^d^	0.18 (−0.28, 0.64)	0.46 (0.09, 0.84)	1.07 (0.71, 1.43)	0.80 (0.37, 1.22)	0.057 (−0.007, 0.121)	0.081
Model 2^g^	−0.08 (−0.96, 0.80)	0.11 (−0.69, 0.90)	0.55 (−0.26, 1.36)	0.28 (−0.70, 1.25)	0.018 (−0.054, 0.090)	0.626

a *Q1 represents the largest decrease and Q4 represents the largest increase in percentage of energy from added sugar (EAS%) intake in each 5-year time interval. Q1 includes participants who had the largest decrease in intake (Q1) in the first, second and third 5-year time interval, Q2 includes those who were in Q2 in the first, second and third 5-year time interval, Q3 includes participants who were in Q3 in the first, second and third 5-year time interval and Q4 includes those who had the largest increase (Q4) in the first, second, and third 5-year time interval*.

b *Estimated change in body weight per 1% increase in EAS% intake*.

c *P for trend was assessed by using change in EAS% intake as a continuous variable*.

d *Model 1: GEE analysis adjusted for gender and initial age*.

e *Model 2: GEE analysis adjusted for gender, initial age, weight, diabetes status, EAS%, and both initial and change in each time interval for fiber intake, total energy intake, glycaemic index, physical activity, and smoking status*.

f *Model 2: GEE analysis adjusted for gender, initial age, weight, diabetes status, EAS% from beverage food sources and both initial and change in each time interval for fiber intake, total energy intake, glycaemic index, physical activity and smoking status*.

g *Model 2: GEE analysis adjusted for gender, initial age, weight, diabetes status, EAS% from non-beverage food sources and both initial and change in each time interval for fiber intake, total energy intake, glycaemic index, physical activity, and smoking status*.

## Discussion

The availability of cohort data provided an opportunity to explore the longitudinal associations between changes in EAS% intake, EAS% intake from both beverage and non-beverage sources and changes in weight over a 15-year period. In this prospective cohort, changes in EAS% intakes were not significantly associated with changes in body weight, regardless of whether the source of AS came from beverages or non-beverages.

Changes in the total energy intakes will result in body weight change (if energy expenditure remains unchanged). During all BMES time intervals, changes in the EAS% intakes were not accompanied by substantial changes in total energy intake (mostly <260 kJ across quartiles). By definition, changes in EAS% intakes were accompanied by compensatory changes in intakes of other macronutrient energy sources, such as fat, protein, alcohol, and other carbohydrates. The AS intake can result in weight gain if it contributes to excess energy intake; however, its isocaloric replacement by other macronutrient sources of energy may not result in weight change (6, 17).

Our results support the concept that when dietary changes are required, the focus should be on a range of macronutrients and overall energy intake, rather than one single nutrient. Recommendations for AS consumption suggest reduction from 10% energy to 5% energy (11, 41), however, this recommendation should consider the possibility that people may consume other macronutrient sources to compensate for the energy reduction. Our findings suggest that those who decreased their EAS% intake by 5% replaced this with increases in energy intake from other macronutrients, such as protein, fat, alcohol, and other carbohydrates. Of course, AS replacement by some nutrients, particularly protein, would provide other health benefits, such as the maintenance of muscle mass and strength (42) but replacement by alcohol may not be preferable.

A cohort study that used baseline AS intake and its food sources to investigate the association with weight gain over time reported different findings (20) compared to our study. High free sugar intake at baseline was associated with significant weight gain in Japanese men during 10 years of follow-up (20). Japanese participants in the highest quartile of free sugar intake had a weight gain of 0.20 kg over 10 years (20). Although the weight gain for the highest quartile of change in EAS% in our study was 0.15 kg, it was not statistically significant. It is worth mentioning that in both studies, this small weight gain over a long period of time may not be clinically significant.

The non-significant results for the association between AS intake and body weight in the BMES cohort is consistent with findings from studies on AS intake and BMI (19, 21). Lee et al. (21) found no significant association between changes in AS and BMI, but reported significant findings for increases in waist circumference (WC), and reported a significant positive association between AS from beverage sources and WC. The major sources of AS intake in the BMES population were non-beverage food sources (e.g., sugar, sweet spreads, and confectionary) (31). However, our non-significant findings for the association between changes in AS intake from non-beverage sources and body weight change in the BMES population is consistent with other studies on solid AS food sources and indicators of obesity (19, 23). It is worth mentioning that in the first 5-year time interval, BMES participants experienced weight gain across the quartiles of change in EAS% sources, however, in the third time interval there was an overall weight loss which could be due to the nature of the aging cohort and the expected body composition changes in the older stages of life (43).

Regarding the beverage AS food sources, most studies have focused on sugar-sweetened beverages. Several cohort studies reported that increased intake of sugar-sweetened beverages was associated with obesity indicators (e.g., weight gain and increases in BMI or WC) (22, 23, 44). AS provided by liquid food sources may play a more powerful role in the development of obesity than AS from solid food sources. This is because the consumption of liquid AS food sources appears to have a weaker satiety effect and may not result in compensation for energy intake to the same extent as solid AS food sources (45), leading to excess food consumption and resultant weight gain. There is also some evidence that carbon dioxide in carbonated beverages (i.e., soft drinks) increases the secretion of ghrelin, the hunger hormone, leading to hunger stimulation and, consequently, increased food intake and weight gain (46).

In contrast, our findings from older Australians did not support the association between AS intake from beverage sources and weight gain. This may be explained by the limited sugar-sweetened beverage intake and small changes (15.9 g) in the overall intake of these beverages among the BMES cohort of older people. Studies show that an increase in sugar-sweetened beverage intake by more than one drink per day (>40g AS or 600 kJ) is positively associated with indicators of obesity (22, 47). In a study conducted by Schulze et al. (22), frequent consumers of these beverages increased their total energy intake by 358 kcal (1,496 kJ) per day and had a weight gain of 4.7 kg over 4 years. However, in BMES participants, the increase in AS in the highest quartile of EAS% change from sugar-sweetened beverages (2%) was 15.9 g and the increase in total energy intake was 224 kJ. Similarly, a cohort of older Spanish adults also observed no significant association, likely due to the occasional intakes of sugar-sweetened beverages in this age group (18).

In BMES participants, the average BMI was <30 kg/m^2^ and the average EAS% was <10%. Therefore, our findings may not be generalizable to populations with high rates of obesity or those with high EAS% intake. Nonetheless, since both the BMI and EAS% intake of BMES participants are similar to national Australian older population (48), our findings may be generalizable to the older Australians. Our study has several strengths, which includes a long follow-up period and a relatively large sample size. However, despite the relatively large sample, we cannot rule out the possibility of lack of sufficient statistical power to detect the significant body weight change as this study was based on the secondary analysis of an existing cohort. Other advantages of this study include dietary assessment at each 5-year follow-up period, accompanied by measurement of body weight (i.e., not self-reported). Moreover, a consistent systematic method was used to estimate the AS content of foods in each time point.

We acknowledge that the possibility of recall bias and the over- or under-reporting of food intake (a limitation of most dietary assessment methods) cannot be ruled out. Nonetheless, implausible intakes were excluded from the dataset and changes in intake were investigated in the same participants during the 15 years, hence any over- or under-reporting may have had a minimal effect on the change in intake, and presumably any bias that may exist would have been consistent over time. Additionally, it is possible that the change in the EAS% categories reflects a statistical phenomenon of regression to the mean (49), noting that those people who reduced their EAS% intake the most (Q1) had the highest baseline EAS% intake (13.7%). This also could reflect they have scope to reduce their intake further than people with a more moderate level of baseline EAS% intake. Furthermore, although the analyses were adjusted for several covariates, the possibility of residual confounders cannot be excluded. However, as the analyses were adjusted for both initial and changes in energy intake over time, medical conditions which influence energy intake have generally been accounted for.

In conclusion, our results do not support the association between changes in EAS% intakes and body weight change in the cohort of older Australians during 15 years of follow-up. This result was similar for beverage and non-beverage AS food sources. Our findings suggest that population-level messages specifically targeted to address weight loss by reductions in AS may be less applicable to older Australians who already consume AS at moderate levels. The findings also provide insights into the importance of including multi-faceted population health messages for weight reduction, such as limiting fat (particularly saturated fat) and alcohol intake, along with any recommendations to reduce AS intake.

## Data Availability Statement

The data analyzed in this study are not readily available because of ethical and privacy considerations. Requests to access the datasets should be directed to PM, paul.mitchell@sydney.edu.au.

## Ethics Statement

The studies involving human participants were reviewed and approved by the Sydney West Area Health Services Ethics Committee and the University of Sydney Human Research Ethics Committee. The participants provided their written informed consent to participate in this study.

## Author Contributions

HM conducted the research, estimated added sugar values, analyzed the data, and drafted the manuscript. VF and PM were involved in the collection of original BMES data. All authors critically reviewed the manuscript and approved the final version submitted for publication.

## Conflict of Interest

The authors declare that the research was conducted in the absence of any commercial or financial relationships that could be construed as a potential conflict of interest.
