# Supplementary material for: Changes in Added Sugar Intake and Body Weight in a Cohort of Older Australians: A Secondary Analysis of the Blue Mountains Eye Study
Source: Front Nutr. 2021 Mar 1;8:629815. doi: 10.3389/fnut.2021.629815 (PMC7957007; doi:10.3389/fnut.2021.629815)
Supplement: Supplementary file 1 [file Table_1.docx]

**Supplementary Table 1.** Baseline characteristics of BMES 1 participants who provided both dietary and weight data in BMES 2 (paired observation) versus participants without paired observation.

|  | **Participants with paired observation** | **Participants without paired observation** | **P*** |
| --- | --- | --- | --- |
| n | 1713 | 1127 | - |
| Women (n, %) | 978 (57.09) | 612 (54.30) | 0.143 |
| Age, years | 63.8 (8.2) | 67.6 (10.3) | <0.001 |
| Current smoker (n, %) | 199 (11.62) | 189 (16.77) | <0.001 |
| Diabetes (n, %) | 104 (6.07) | 102 (9.05) | <0.001 |
| Married (n, %) | 1186 (69.24) | 697 (61.85) | <0.001 |
| Qualification after leaving school (n, %) | 1040 (60.71) | 582 (51.64) | <0.001 |
| Home ownership (n, %) | 1567 (91.48) | 946 (83.94) | <0.001 |
| Living alone (n, %) | 381 (22.24) | 317 (28.13) | 0.003 |
| Energy, kJ | 8599 (2481) | 8485 (2664) | 0.253 |
| Fat, E% | 32.82 (6.21) | 33.09 (6.45) | 0.270 |
| Protein, E% | 17.76 (3.08) | 17.62 (3.30) | 0.254 |
| Alcohol, E% | 3.81 (5.61) | 3.89 (5.94) | 0.726 |
| Carbohydrate, E% | 46.91 (7.64) | 46.65 (8.53) | 0.407 |
| Added sugar, E% | 9.40 (5.16) | 10.14 (5.61) | <0.001 |
| BMI, kg/m^2^ | 26.22 (4.28) | 26.12 (4.69) | 0.575 |
| Body weight, kg | 72.14 (13.51) | 71.16 (15.32) | 0.080 |

BMI: body mass index

Data were presented as mean (SD) for continuous variables, and *n* (%) for categorical variables.

*All P values were assessed using the Pearson’s chi-squared test except for age, BMI, body weight and dietary data where P values were assessed using the independent t-test.
